# From Guidelines to Action: Tackling Risk Factors for Surgical Site Infections

**DOI:** 10.3390/antibiotics14010040

**Published:** 2025-01-06

**Authors:** Caterina Elisabetta Rizzo, Roberto Venuto, Paola Tripodi, Linda Bartucciotto, Elvira Ventura Spagnolo, Antonio Nirta, Giovanni Genovese, Isabella La Spina, Sabrina Sortino, Alessandro Nicita, Francesco Loddo, Bruno Romeo, Raffaele Squeri, Cristina Genovese

**Affiliations:** 1 Department of Biomedical and Dental Sciences and Morphofunctional Imaging, University of Messina, 98124 Messina, Italy; paola.tripodi92@gmail.com (P.T.); lindabartucciotto@gmail.com (L.B.); elvira.venturaspagnolo@unime.it (E.V.S.); antonio.n2396@gmail.com (A.N.); gigenovese@unime.it (G.G.); isabella.laspina@gmail.com (I.L.S.); sabrinasortino87@gmail.com (S.S.); sandronicita94@gmail.com (A.N.); france.loddo@gmail.com (F.L.); b.romeo@hotmail.it (B.R.); squeri@unime.it (R.S.); crigenovese@unime.it (C.G.); 2 Department of Prevention, Local Health Authority of Messina, 98123 Messina, Italy

**Keywords:** surgical site infections, healthcare, prevention strategies

## Abstract

**Background/Objectives:** Surgical site infections are a serious public health concern, representing a significant burden on healthcare systems and society. Their occurrence is influenced by several factors, including patient demographics, healthcare facilities and the specific circumstances surrounding surgery. The use of prophylactic antibiotics in this context carries both potential benefits and risks. The aim of this study is to investigate potential risk factors that may adversely affect the development of SSIs, as well as to assess the appropriateness and adherence to perioperative antibiotic prophylaxis. **Methods:** This observational study was conducted from October 2023 to October 2024 at the University Hospital of Messina, Italy, a hospital performing both thoracic and vascular surgery. Data were collected using a questionnaire regarding socio-demographic data, risk factors, clinical and surgical data and details regarding the administration of antibiotics. **Results:** This study included 117 patients with an average age of 63 ± 12.36 SD years, 70.9% from the Thoracic Surgery Unit and 29.1% from the Vascular Surgery Unit. The most administered antibiotic was cefazolin, and antibiotic administration time was in compliance with the guidelines. **Conclusions:** Our data show that the implementation of evidence-based guidelines, healthcare professionals’ education and correct antibiotic use can reduce the burden of SSIs by improving patient care.

## 1. Introduction

With a global incidence of approximately 31%, surgical site infections (SSIs) are among the most common healthcare-associated infections (HAIs) [1]. They represent a significant burden on both healthcare systems and society, contributing to increased patient morbidity, prolonged hospital stays, elevated treatment costs and more complex recovery processes, exacerbating healthcare expenditures and reducing overall healthcare efficiency [2]. SSI rates vary across different surgical procedures and healthcare systems, with reported incidences ranging from 2% to over 20% [2,3]: for instance, data from the Italian SSI monitoring program for non-prosthetic surgeries between 2009 and 2011, reported by the SNICh study group, identified SSIs in 1628 cases (2.6%), with 30-day post-discharge surveillance accounting for 60% of SSI diagnoses [4]. This highlights the importance of extended surveillance post-discharge to identify infections that may not present immediately following surgery. Similarly, another study reported an SSI prevalence of up to 11% [5]. This variability in SSI rates calls for further investigation into the factors that contribute to these differences across surgical settings.

The occurrence of SSIs is influenced by a multitude of factors, including patient demographics, healthcare facilities and the specific circumstances surrounding each surgery. Key risk factors, consistently linked to an increased incidence of SSIs, include advanced age (>55 years), diabetes mellitus (especially poorly controlled hyperglycemia during the perioperative period), immunocompromised states (such as HIV or patients on immunosuppressive therapy) and the level of surgical expertise (with higher infection rates observed in procedures performed by junior residents compared to senior surgeons). Other risk factors are represented by the nature of the surgery (emergency procedures), drain placement, wound classification (highest in contaminated or dirty wounds), type of closure (multilayer closure), prolonged hospital stays, extended surgery duration (>2 h) and specific types of surgery (e.g., cholecystectomy) [6]. Other surgical-related factors, such as the use of invasive devices, perioperative blood loss and the complexity of the intervention, are also critically important [7,8]. In addition, obesity, smoking and comorbidities are significant patient-related risk factors [9,10,11]. Healthcare system-related factors, such as inadequate adherence to infection control protocols, inappropriate antibiotic prophylaxis and insufficient sterilization practices, further contribute to SSI incidence [12].

Infections that lead to SSIs primarily arise from exogenous sources or the patient’s endogenous skin microbiota [13]. Among the most common Gram-positive bacteria causing SSIs are methicillin-sensitive *Staphylococcus aureus* (MSSA) and methicillin-resistant *Staphylococcus aureus* (MRSA), as well as coagulase-negative staphylococci [14]. These pathogens exhibit persistence and virulence at surgical sites, largely due to their ability to form biofilms and their resistance to antibiotics, complicating both treatment and prevention efforts [15]. Additionally, abdominal and urinary tract infections associated with SSIs are often linked to Gram-negative bacteria, particularly *Escherichia coli* [16]. The preeminent role of *E. coli* in these infections is concerning, as it is frequently associated with the development of extended-spectrum beta-lactamases (ESBLs), which contribute to growing antibiotic resistance [13]. Furthermore, infections caused by Enterococcus species, particularly *Enterococcus faecalis* and *Enterococcus faecium*, pose a growing challenge due to their innate resistance to multiple classes of antibiotics, including vancomycin [14,15].

The use of prophylactic antibiotics in this context carries both potential benefits and risks. The decision to use antibiotics should be based on the patient’s individual risk factors, including both procedure-related and patient-related considerations, as well as the potential severity of an SSI and the efficacy of prophylaxis for the specific type of surgery. Moreover, the potential adverse effects of prophylactic antibiotics, such as an increased risk of *Clostridium difficile*-associated colitis or diarrhea, should be carefully weighed.

These findings reinforce the complexity of SSI prevention, as multiple interacting factors influence infection risk. Therefore, SSI prevention necessitates a comprehensive, multifaceted strategy. Key measures include optimizing the patient’s condition prior to surgery, adhering strictly to sterile techniques during surgery, administering appropriate antibiotic prophylaxis, maintaining rigorous hand hygiene, performing aseptic wound care and enhancing surveillance throughout the perioperative period [12]. Notably, SSI rates have been significantly reduced by implementing interventions such as those recommended by the Healthcare Infection Control Practices Advisory Committee (HICPAC), which developed a comprehensive package for preventing HAIs that includes aseptic techniques, skin decontamination and appropriate antibiotic administration [16]. Additionally, in Italy, national health standards for SSI prevention, including prophylactic antibiotic administration before and after surgery, were enacted through the Gelli–Bianco law [17]. The importance of these prophylactic measures cannot be overstated, as they are fundamental to reducing the incidence of SSIs.

Based on these assumptions, the aim of this study is to investigate potential risk factors that may adversely affect the development of SSIs, as well as to assess the appropriateness and adherence to perioperative antibiotic prophylaxis in a southern Italian University Hospital. Through a detailed examination of local practices and the identification of key risk variables, this research seeks to provide valuable insights for improving SSI prevention and management in the regional healthcare setting.

## 2. Results

This study included 117 patients, with the majority (70.9%, *n* = 83) from the UHM “Thoracic Surgery Unit” and 29.1% (*n* = 34) from the UHM “Vascular Surgery Unit”. The participants’ average age was 63 ± 12.36 SD years.

Table 1 provides a summary of the patients’ socio-demographic information, including age, gender and other pertinent factors, whereas Table 2 shows the distribution of the study sample according to clinical data.

In 6.8% of cases, the patient’s recovery was preceded by the occurrence of a community-acquired infection.

Regarding individual risk factors, about three-quarters (*n* = 59, 50.4%) of the sample had a BMI ≥ 25, 33.3% (*n* = 39) were smokers, and 22.2% (*n* = 26) had diabetes; in addition, a prolonged hospital stay (>15 days) was reported by 11.7% (*n* = 10) of the sample; the stay at the vascular surgery ward’s length was 9.44 ± 10.24 SD days, while at the thoracic surgery ward, it was 8.89 ± 9.92 SD days.

**Figure 1:**
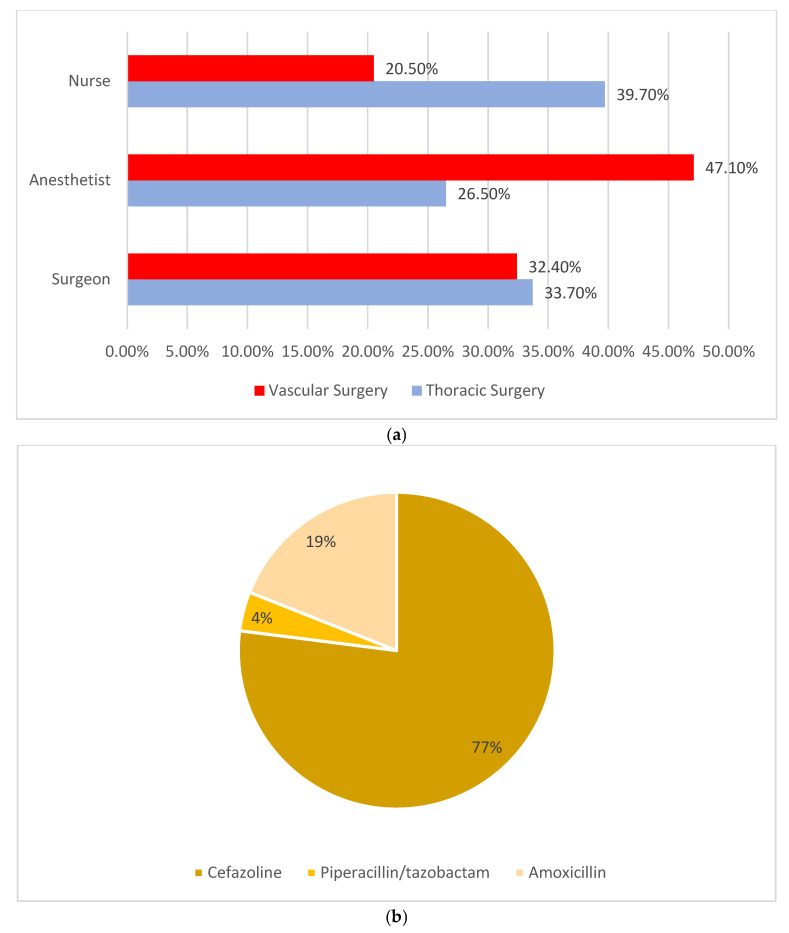
Liability of administration of antibiotics by ward and HCWs (**a**) and type of antibiotics administered (**b**).

As regards antibiotic use, cefazolin was the primary class of antibiotics given in accordance with National Guideline System (SNLG) indications (see Figure 1b); in addition, the anesthetists bore the majority of the healthcare liability for administering antibiotics (see Figure 1a) [18].

Antibiotics were administered prior to surgery in 40.2% (*n* = 47) of cases, while 43.6% (*n* = 5) received them postoperatively; intraoperative antibiotic prophylaxis was administered only in 2.6% (*n* = 3) of procedures.

Therefore, antibiotic administration timing was in compliance with the guidelines.

Statistical associations between the timing of antibiotic administration and qualitative characteristics are shown in Table 3.

Males were more likely than females to receive antibiotics postoperatively; however, there was no statistically significant difference in the overall amount of antibiotics administered (preoperatively, intraoperatively and postoperatively).

About 17.6% (*n* = 9) of patients with diabetes received postoperative antibiotics; however, our study did not find a statistically significant difference.

Postoperative antibiotic therapy was administered to 19.6% (*n* = 10) of patients on immunosuppressive therapy and 80.4% (*n* = 41) of non-immunosuppressed patients, demonstrating a statistically significant relationship between preoperative immunosuppressive therapy and postoperative antibiotic administration (*p* = 0.001).

Furthermore, there was a significant difference in the types of surgical interventions coded using ICD-9-CM before the surgical procedure was administered (*p* = 0.049) for video endoscopy (21.3%), drainage (27.7%), angioplasty (36.2%), lung resection (10.6%) and biopsies (4.2%).

There was a significant difference between intraoperative and preoperative antibiotic administration (*p* = 0.005), especially in relation to preoperative administration before surgical procedure (*p* = 0.001), depending on the procedure type (emergency or elective).

Another statistically significant correlation was found between the ASA score and preoperative administration (*p* < 0.001). Finally, there was a significant correlation between immunosuppressive therapy and the postoperative administration of antibiotics (*p* < 0.001).

## 3. Discussion

The results of our study show that in all cases, the administration of antibiotics fits the local guidelines.

Gender disparities at the beginning of SSIs are rarely studied in research. The risk of SSIs in abdominal surgery was shown to be lower in women than in men, while in cardiac surgery, women had a greater incidence of surgical site infections (5.50 vs. 3.02; *p* < 0.001), but there were no gender-specific differences in orthopedic or vascular surgery [19,20].

Moreover, the relationship between diabetes and a higher incidence of SSIs, as well as the possible connection between hyperglycemia and SSIs, has been extensively documented in the media in recent years [21,22]. In our study, we found a statistical correlation between diabetes and the use of perioperative antibiotic prophylaxis; in the scientific literature, several studies confirm this finding and, in particular, a recent meta-analysis revealed a strong correlation between diabetes and SSIs that held true after adjusting for BMI and across a variety of surgical procedures; in addition, meta-analyses of trials adjusted for hyperglycemia still show that diabetes history is a substantial risk factor [23].

Numerous inflammatory and autoimmune diseases are treated with chronic steroid therapy, although its side effects are well-known. Regarding the role that continuous steroid therapy plays in predisposing patients to perioperative problems, there is still a gap in the literature. Chronic preoperative steroid medication has been shown by Ranson et al. to be an independent risk factor for perioperative complications, including wound dehiscence, urinary tract infection, pulmonary embolism, readmission and nonhome release. Patients who are excessively obese are even more at risk [24].

ICD-9-CM diagnosis codes for SSIs have not yet been extensively studied [25]. However, in our study, when comparing the type of surgical intervention, coded using ICD-9-CM, statistical significance was found for preoperative administration, particularly for video endoscopy, drainage, angioplasty, lung resection and biopsies. Analogous results were reported in a research by Onyekwelu et al., where the association between Surgical Wound Classification (SWC) and SSI development is not statistically significant (*p* > 0.005) [26]. This may indicate that the model has predictive value for future SSIs, which was not its initial intended application.

Moreover, the scientific literature shows a significant association between the rate of SSIs and ASA classification [27], with some authors reporting that ASA class 1 decreased the risk for SSIs by 0.3 times compared to ASA 3 [28].

Additionally, in patients undergoing prolonged antibiotic treatments, it is certainly less likely to isolate bacteria from the wound swab [29]. In a previous study, the same association was evaluated in patients undergoing cardiac surgery, showing that a longer duration of prophylaxis does not alter the percentage of SSIs even in the long term [30].

A recent observational study conducted on patients undergoing cemented hip arthroplasty shows a lower number of reoperations for removal or replacement of the prosthesis when prophylaxis is continued for 24 h and an antibiotic is added to the cement [31]. Three recent studies, one conducted on patients undergoing appendectomy (for non-perforated appendicitis), one on patients undergoing surgery for gastric carcinoma, and a third on patients undergoing gynecological surgery, confirm that the administration of a single perioperative dose of antibiotic has the same effect in preventing surgical site infections as repeated doses [32]. There is no evidence that continuing antibiotic prophylaxis in the presence of a drain reduces postoperative infectious complications.

Regarding the type of procedure, whether emergency or elective, in our study, there was a significant difference in preoperative and intraoperative antibiotic administration, particularly in association with preoperative administration; the finding that administration of antibiotics could impact the incidence of SSIs is well-known [33].

Finally, the time of administration was adherent to the guidelines (43.6% of the antibiotic administration occurred 60 min after the intervention), confirming the literature results [34].

### Limitations of This Study

The limitations of this study are that it is an observational epidemiological study and, therefore, exposure is not controlled by the investigator. Observational studies are by far the most common form of clinical research because of their relatively low complexity, cost and ethical constraints compared to randomized trials or other forms of clinical experimentation. Bias, confounding and issues with validity are more common in observational studies. The results could be affected by several biases, for instance, selection bias and social desirability bias.

## 4. Materials and Methods

This study was conducted from October 2023 to October 2024 at the University Hospital “G. Martino” of Messina (UHM), Italy, a hospital performing both thoracic and vascular surgery. This retrospective observational study aimed to evaluate whether perioperative antibiotic prophylaxis adhered to guidelines in patients undergoing these types of surgeries.

Data were collected using an ad hoc questionnaire that was specifically developed for this study. The questionnaire was composed of the following four sections:

(a) Socio-demographic data (age, gender and BMI);
(b) Risk factors (smoking, diabetes and immunosuppressive therapy);
(c) Clinical and surgical data, including ongoing infections, ICD9-CM codes, whether the surgery was urgent or elective, the use of video-endoscopic techniques, surgery classification, duration, the ASA Physical Status Classification System score and the use of blood transfusion or derivatives;
(d) Details regarding the administration of antibiotics, including the person responsible for their administration, type of antibiotic and time of administration, whether before, during or after the surgical procedure.

The questionnaire was developed in Italian, as the study population predominantly spoke Italian, and was validated through a pilot phase where a small sample of the target population was tested for clarity and consistency. Following validation, the questionnaire was administered to all eligible patients during their hospital stay. The target population included patients undergoing elective or urgent thoracic or vascular surgeries, with a focus on those at risk for SSIs due to various pre-existing conditions.

The inclusion criteria for participation were as follows:

(1) patients undergoing thoracic or vascular surgery at the hospital during the study period
(2) who provided informed consent to participate and
(3) were aged 18 or older.

The exclusion criteria were as follows:

(1) patients under 18
(2) who refused to participate in the study
(3) with severe cognitive impairments or language barriers that would hinder survey completion and
(4) with no available preoperative antibiotic administration data.

In order to test the hypotheses, contingency tables were created using the chi-squared (χ^2^) test. The approach of partitioning the degrees of freedom was applied exclusively in the hypothesis of rejection of the null hypothesis (H0) and in the presence of r × k tables. The synthetic and inferential statistical analyses were performed using R software version 4.4.0.

## 5. Conclusions

Surgical site infections remain a significant challenge in healthcare settings. Patients who develop surgical site infections often require further surgical interventions, experience delayed wound healing and endure long-term disabilities [35]. The costs associated with SSIs are staggering [36,37,38,39], with direct medical expenses including prolonged hospital stays, additional surgical procedures and expensive antibiotic regimens [40]; furthermore, indirect costs such as lost productivity, rehabilitation and long-term care exacerbate the economic impact [41]. The implementation of evidence-based guidelines, healthcare professionals’ education and correct antibiotic use can reduce the burden of SSIs by improving patient care. Further research is needed to investigate new preventive strategies and therapeutic interventions to reduce the burden of SSIs.

Antibiotic resistance, which undermines the effectiveness of antibiotics, is making once-treatable infections more difficult to manage [42]. It has become a global public health threat, contributing to higher morbidity, mortality and healthcare costs. Monitoring and understanding the spread of antibiotic-resistant bacteria is crucial for effective control and prevention measures [43]. Since its initial edition, the Italian National Plan to Fight Antimicrobial Resistance (in Italian Piano nazionale di contrasto all’antibiotico-resistenza, PNCAR) saw the consolidation of HAI surveillance, which included SSIs [44]. Surveillance systems play a vital role in the early detection, evaluation and response to antibiotic resistance by tracking the prevalence of specific antibiotic-resistant strains, such as extended-spectrum β-lactamase (ESBL)-producing *Escherichia coli* [45]. In response to this challenge, the World Health Organization (WHO) introduced the AWaRe classification system as a tool to promote responsible antibiotic prescribing practices [46]. The Access, Watch and Reserve (AWaRe) classification system is an evidence-based approach designed to guide healthcare professionals in the appropriate use of antibiotics. 

Despite significant advancements in antimicrobial stewardship and surveillance, much work is still required.

First of all, ongoing research is needed to explore novel preventive measures, improve the accuracy and timeliness of diagnostic tools and develop new therapeutic interventions for infections caused by resistant pathogens. Concerning that point, effective alternative preventive and therapeutic measures have long been known but have been overlooked for an extended period, mainly due to the lack of commercial impact of these simple solutions. Notably, the effectiveness of inexpensive oxidizing agents like hydrogen peroxide [47] and chlorine dioxide [48] for safe and efficient disinfection is well-documented. These substances also show promise in treating infectious pathogens therapeutically, similar to other effective treatments, such as high-dose ascorbic acid for combating sepsis and other infections or intoxications [49]. In fact, there is substantial potential in high-dose ascorbic acid therapy, as supported by decades of research. Yet, it remains largely ignored by both the current healthcare system and academia [50,51].

Additionally, healthcare systems must continue to improve surveillance efforts to track the emergence of new resistant strains, enabling the rapid implementation of control measures. Continued investment in antimicrobial resistance research, along with the implementation of comprehensive stewardship initiatives, will be critical to reducing the burden of antimicrobial resistance and preserving the effectiveness of antibiotics. Furthermore, antimicrobial stewardship programs can play a crucial role in reducing antimicrobial resistance overall [52]. By improving prescribing practices and promoting appropriate antibiotic use, these programs aim to prolong the effectiveness of existing antimicrobial agents. 

In summary, the evidence presented in this research suggests that the effective implementation of stewardship interventions can optimize antibiotic use, minimize patient harm and preserve antibiotic efficacy for future generations. Recent study findings emphasize the need for ongoing research, enhanced surveillance systems and sustained efforts to implement and evaluate antimicrobial stewardship initiatives to address the global threat of antimicrobial resistance [53]. 

## Figures and Tables

**Table 1:** Distribution of the study sample according to socio-demographic data and risk factors.

	*n* (117)	%
Gender		
Male	85	72.6
Female	32	27.4
Body mass index		
<17	4	3
18–24	35	29.9
25–29	44	37.6
>30	15	12.8
Unknown	19	16.7
Smoking habit		
Smokers	39	33.3
Non-smokers (including ex-smokers)	66	56.4
Unknown	27	10.3
Diabetes		
Yes	26	22.2
No	91	77.8
Immunosuppressive therapy		
Yes	11	9.4
No	106	90.6

**Table 2:** Distribution of the study sample according to clinical and surgical data.

	*n* (117)	%
Clinical infection at admission		
Yes	8	6.8
No	109	93.2
Type of operation (NAS-NCR)		
Clean	18	15.4
Clean–contaminated	97	82.9
Contaminated	2	1.7
Procedure type		
Elective	88	75.2
Emergency	28	23.9
Unknown	1	0.9
Use of blood transfusion or derivatives		
Yes	5	4.3
No	101	86.3
Unknown	11	9.4
Prosthesis implants		
Yes	8	6.8
No	102	87.2
Unknown	7	6.0
Video endoscopy		
Yes	53	45.3
No	63	53.8
Unknown	1	0.9
ASA score		
1	4	3.4
2	31	26.5
≥3	82	70.1

**Table 3:** Administration of antibiotics before, during and after surgical procedure versus qualitative characteristics (significant *p*-values are reported in bold).

Variable	Antibiotic Administration Before Surgery	*p*-Value	Antibiotic Administration During Surgery	*p*-Value	Antibiotic Administration After Surgery	*p*-Value
Total % (*n*)	40.2% (47)		2.6% (3)		43.6% (51)	
Gender						
Male	68.1% (32)	0.662	66.7% (2)	0.851	68.6% (35)	0.391
Female	31.9% (15)		33.3% (1)		31.4% (16)	
Diabetes						
Yes	21.3% (10)	0.740	33.3% (1)	0.326	17.6% (9)	0.295
No	78.7% (37)		66.7% (2)		82.4% (42)	
Immunosuppressive therapy						
Yes	12.8% (6)	0.510	0% (0)	0.356	19.6% (10)	**0.001**
No	87.2% (41)		100% (3)		80.4% (41)	
ICD9 CM Code						
Fiberoptic endoscopy	21.3% (10)	**0.049**	33.3% (1)	0.733	21.6% (11)	0.076
Drainage	27.7% (13)		33.3% (1)		33.3% (17)	
Angioplasty	36.2% (17)		33.3% (1)		27.5% (14)	
Lung resections	10.6% (5)		0% (0)		9.8% (5)	
Biopsies	4.3% (2)		0% (0)		7.8% (4)	
Type of surgical procedure						
Emergency	34% (16)	**0.001**	33.3% (1)	**0.005**	19.6% (10)	0.346
Elective	63.8% (30)		66.7% (2)		78.4% (40)	
Unknown	2.1% (1)		0% (0)		2% (1)	
Classification of surgical procedure						
Clean	12.8% (6)	0.280	0% (0)	0.928	9.8% (5)	0.337
Clean–contaminated	87.2% (41)		100% (3)		88.2% (45)	
Contaminated	0% (0)		0% (0)		2% (1)	
Prosthesis implant						
Yes	17% (8)	**0.001**	0% (0)	0.418	3.9% (2)	0.364
No	80.9% (38)		100% (3)		92.2% (47)	
Unknown	2.1% (1)		0% (0)		3.9% (2)	
Blood transfusion						
Yes	8.5% (4)	**0.001**	0% (0)	**0.001**	7.8% (4)	0.060
No	87.2% (41)		100% (3)		88.2% (45)	
Unknown	4.3% (2)		0% (0)		3.9% (2)	

## Data Availability

Data is contained within the article.
